# Sentinel node lymphocytes: tumour reactive lymphocytes identified intraoperatively for the use in immunotherapy of colon cancer

**DOI:** 10.1038/sj.bjc.6603126

**Published:** 2006-04-25

**Authors:** P Marits, M Karlsson, K Dahl, P Larsson, A Wanders, M Thörn, O Winqvist

**Affiliations:** 1 Department of Internal Medicine, Uppsala University Hospital, Uppsala University, 75185 Uppsala, Sweden; 2 Department of Medicine, Unit of Clinical Allergy Research, Karolinska Hospital, Karolinska Institutet, 17176 Stockholm, Sweden; 3 Department of Surgery, South Stockholm General Hospital, Karolinska Institutet, 11883 Stockholm, Sweden; 4 Department of Pathology, Uppsala University Hospital, Uppsala University, 75185 Uppsala, Sweden

**Keywords:** sentinel node, colon cancer, adoptive immunotherapy, tumour reactive lymphocytes

## Abstract

The sentinel node is the first lymph node to receive lymphatic drainage from a tumour and is usually the first site of metastases. Today, the sentinel node is used for tumour staging. Here, we focus on its immunological role and investigate lymphocytic function in sentinel nodes, identified intraoperatively by peritumoural dye injection, from 15 patients with colon cancer. Tumour infiltrating lymphocytes, sentinel and nonsentinel lymph node cells and peripheral blood leukocytes were studied by flow cytometry, proliferation assays and interferon-*γ* secretion after activation with autologous tumour homogenate. Whereas tumour-infiltrating lymphocytes were nonresponsive in the proliferation assays, lymphocytes from sentinel nodes proliferated dose dependently and secreted interferon-*γ* upon stimulation with tumour homogenate. The responses were of varying magnitude and tended to be weaker in metastatic sentinel nodes. Sentinel node lymphocytes represents an enriched source of tumour reactive lymphocytes, and may be useful in future trials of adoptive immunotherapy.

Colorectal cancer (CRC) is a major cause of morbidity worldwide, accounting for over 1 million new cases and 500 000 deaths per year (Parkin *et al*, 2005). Surgery is the primary treatment modality and is curative in approximately 50% of patients, however, the remaining half of patients will ultimately die of metastatic disease (Obrand and Gordon, 1997). Standard, Fluorouracil-based, chemotherapy in the adjuvant setting confers at most 10% absolute increase in survival in patients with lymph node positive disease (Duke's C) (Arkenau *et al*, 2003), while there is no evidence for its benefit in patients with localised disease (Benson *et al*, 2004). Thus, there is a great need for developing alternative adjuvant treatments.

Studies of prognostic factors in colorectal cancer point out the histological status of the regional lymph nodes as the most important predictor of survival (Swanson *et al*, 2003) and the presence of regional lymph node metastases implies a 40–50% reduction in 5-year survival rates. The sentinel node is defined as the first lymph node, or nodes, to receive lymphatic drainage from a tumour. It can be identified during surgery by injection of a tracer substance around the tumour. The tracer is transported in the lymph capillaries and accumulates in the sentinel node(s), thus identifying the tumour draining lymph node. The histological status of the sentinel node is regarded as representative for the status of the entire lymphatic field. The sentinel node method, first introduced in 1977 (Cabanas, 1977), is established in malignant melanoma and breast cancer (Leong, 2004) and it has also been applied to colon cancer (Saha *et al*, 2000; Thörn, 2000). Recently, we demonstrated the usefulness of sentinel node detection for the staging of colon cancer (Dahl *et al*, 2005).

According to the immune surveillance hypothesis (Burnet, 1970), the immune system is continuously sensitised against developing tumours, most of which are eliminated at an early stage. Strong experimental evidence in favour of this notion has been presented in recent years, extensively reviewed by Dunn *et al* (2004). The identification of specific tumour antigens has created new possibilities for tumour immunotherapy (Rosenberg, 2001) and many immunotherapeutic approaches are now being translated into clinical trials (Ribas *et al*, 2003). Among these, adoptive transfer of tumour antigen-specific lymphocytes seems particularly promising. These attempts are usually based on either mononuclear cells from peripheral blood or tumour infiltrating lymphocytes (TIL) separated from fresh tumour specimens. In recent trials, treatment of patients with malignant melanoma with autologous transfer of expanded TILs, objective response rates of up to 51% has been reported (Rosenberg and Dudley, 2004). In CRC, the presence of TIL correlates with a more favourable prognosis (Giorgio *et al*, 1992) and TIL from CRCs, expanded *in vitro* in IL-2, display specific cytokine secretion in response to autologous tumour cells (Hom *et al*, 1993). However, TIL from several human tumours, including CRC, display reduced levels of the CD3 zeta chain (Nakagomi *et al*, 1993). In addition, T-cell responses to mitogenic stimulation are decreased in lymphocytes from the venous blood draining CRCs, as compared with lymphocytes obtained from the arterial blood supplying the tumour (Rigg *et al*, 1991). Thus, the limited number of TIL that can be obtained from solid tumour biopsies may not be an ideal cell population for adoptive immunotherapy.

As experimental evidence indicate that activation of naïve T cells occurs within the highly specialised microenvironment of secondary lymphoid organs, that is lymph nodes and spleen (Itano and Jenkins, 2003), the sentinel node may be regarded as the primary site for the immune system to encounter tumour antigens. In the present study, we take advantage of the specific sentinel node detection to investigate the immunological tumour reactivity in sentinel lymph nodes draining human colon cancers.

## MATERIALS AND METHODS

### Patients

Fifteen patients with colon cancer, with no signs of distant metastases or lymph node involvement prior to surgery, seven men and eight women, with an average age of 71 years were included in the study (Table 1). The study was approved by the local ethical committee and informed consent was given by the patients.

### Identification of sentinel nodes

The colonic tumour site was mobilised through division of peritoneal adhesions to facilitate inspection of tumour and mesentery. One ml Patent blue dye (Guerbet, Paris) was injected superficially in the serosa around the tumour. Within 5 min, one to four blue-coloured mesenteric lymph nodes were identified macroscopically as sentinel nodes and they were marked with sutures.

### Preparation of specimens

The sentinel and nonsentinel nodes were cut in half. Slices <1 mm thick were cut from the central and the peripheral part of the nodes for flow cytometry and proliferation analysis. The rest of the node underwent routine histopathological examination. Tumours were histopathologically classified as Duke's stages A–D (Duke, 1932) (Table 1). One piece of the primary tumour (including part of the invasive margin) was removed for flow cytometry analyses and as an antigen source.

### Flow cytometry analyses

Peripheral blood leukocytes (PBL), lymph node cells and tumour cell suspensions at 1 × 10^6^ cells sample^−1^ were subjected to investigation using flow cytometry (FACS). Cells were washed in PBS containing 2% FCS and 0.05% NaN_3_ and stained with fluorophore conjugated antibodies against the cell surface molecules CD4 PE, CD8 PerCp (Becton Dickinson, San Jose, CA, USA) and the very early activation marker CD69 FITC (Pharmingen, San Jose, CA, USA). After staining, cells were investigated using a FACSCalibur (Becton Dickinson) and data were analysed using the Cellquest computer software (Becton Dickinson).

### Immunological evaluation

Single cell suspensions from lymph nodes and tumours were obtained by gentle pressure using a loose fit glass homogeniser. Peripheral blood leukocytes were purified by ficoll-paque (Pharmacia, Amersham, Uppsala, Sweden). Cells were resuspended and washed twice in RPMI 1640 (Life technologies, Rockville, MD, USA) containing 2.5% fetal calf serum (FCS) (Life technologies). Finally, cells were resuspended in RPMI 1640 proliferation media containing 10% human AB serum (Sigma-Aldrich, St Louis, MO, USA), 1% penicillin–streptomycin (Sigma) and 1% glutamine (Sigma). Tumour samples were homogenised using a Ultra-turrax in 5 v (w v^−1^) of 2 × PBS followed by 5 min denaturation at 97°C. Tumour homogenates were diluted to 1 : 10 and 1 : 100 in complete proliferation media. Purified PBL and lymph node cells were used at 3 × 10^5^ cells well^−1^ in time course proliferation assays against diluted tumour homogenate, Con A 10 *μ*g ml^−1^ (Sigma) or carcinoembryonic antigen 100 *μ*g ml^−1^ (Sigma) in triplicate. Proliferation was measured by adding 1 *μC*_i_ of ^3^H-Thymidine/well (Amersham) 18 h prior to harvesting. Samples were subjected to scintillation counting.

Stimulations for the measurement of IFN-*γ* secretion were performed in 96-well plates with 3 × 10^5^ cells well^−1^ in triplicate with tumour homogenate diluted 1/10 and 1/100, or Con A 10 *μ*g ml^−1^ (Sigma). The amount of secreted IFN-*γ* was measured with ELISA (Human IFN-*γ* Duoset, R&D Systems) on culture supernatants in pooled samples of the triplicates.

## RESULTS

### Intraoperative identification of sentinel node and pathological classification

**Figure 1:**
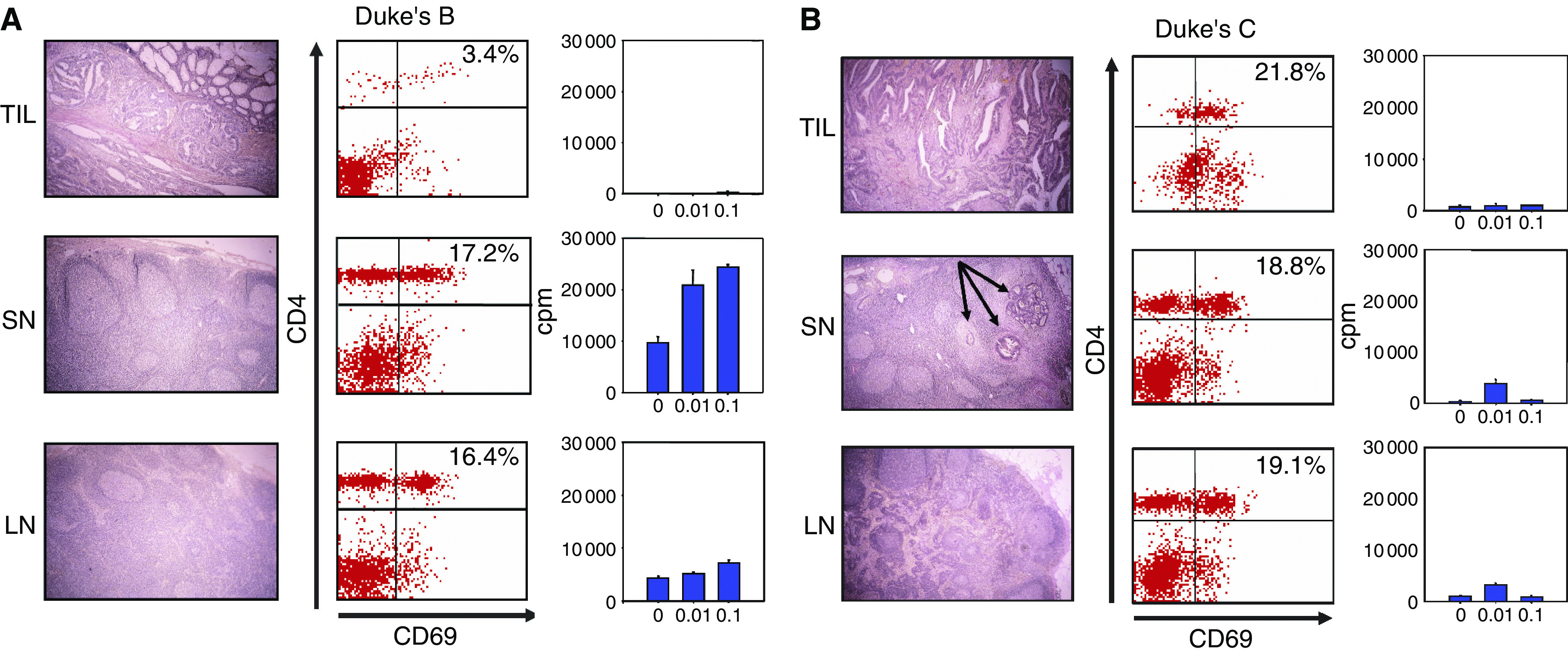
Characterisation of lymphocytes. In 15 colon cancer patients, tumour-draining, sentinel lymph nodes were identified by peritumoural injection of a blue tracer. Specimens from the tumour (CC), sentinel node (SN) and nonsentinel node (LN) and stained with haematoxylin–eosin (left panels) (40 ×). Data from patient no 1 with a Duke's B tumour (**A**) and patient no 12 with Duke's C (**B**). Arrows indicate the presence of metastatic colon cancer cells in a sentinel node (**B**, left panel, SN). Single cell suspensions from the specimens were stained with antibodies against CD4 and the activation marker CD69 and analysed using flow cytometry (middle panels), the percentage of double positive activated CD4 T-helper cells are indicated in the upper right corner. Cell suspensions in triplicates were incubated with a 10- and 100-fold dilutions of autologous tumour homogenate in a day 5–7 proliferation assay. Cells were pulsed 18 h before harvesting with 1 *μC*_i_ 3H-Thymidine. Proliferation data from day 5 (**A**) and day 6 (**B**), respectively, are shown. Error bars indicate s.e.m. (right panels).

One to four sentinel nodes (average 2.3) were detected intraoperatively using Patent blue injection in the circumference of the tumour. Patient characteristics and location of the tumours are presented in Table 1. Upon macroscopical dissection of the removed specimens, between five and 29 lymph nodes (average 19) were identified and embedded for histopathological evaluation (Figure 1A and B, left panels). Nine of the patients (Table 1) showed no signs of metastatic spread to sentinel node(s), nor to other lymph nodes despite that tumours grew through the bowel muscular wall (Figure 1A left panel) and they were classified as Duke's B. Patients no 10–14 (Table 1) had metastatic spread to the sentinel node and were histopathologically classified as Duke's C (Figure 1B left panel). Between one and six metastatic lymph nodes were identified by histopathology (Table 1). Patient no 15 in addition to one metastatic sentinel node had distant metastases to the liver, found at surgery, and was consequently classified as Duke's D (Table 1).

### Characterisation of immune cells in sentinel nodes

Single cell suspensions of lymphocytes collected from the tumour, sentinel and nonsentinel lymph node specimens, and peripheral blood (PBL), were prepared as described in Material and Methods. The number of sentinel nodes obtained and average cell yield from the sentinel node specimens for each patient are given in Table 1. Cell suspensions were triple stained with antibodies recognising the very early activation marker CD69 and the surface antigens CD4 (Figure 1A and B, middle panels) and CD8 (not shown) followed by flow cytometry analyses (FACS). The lymphocytic gate was established by gating on forward and side scatter characteristics. The percentage of CD4^+^ and CD8^+^ cells in the lymphocytic gate varied between lymph nodes (Table 1). No significant correlation was found between the ratio of CD4^+^ to CD8^+^ cells and tumour stage or proliferative response (data not shown). Similar numbers of activated CD4^+^CD69^+^ lymphocytes were found both in sentinel and nonsentinel nodes regardless of absence (Figure 1A middle panel) or presence of metastases (Figure 1B middle panel). All investigated tumours contained tumour-infiltrating lymphocytes (TILs) to a various extent. Tumour-infiltrating lymphocytes were both of the CD4 and CD8 subsets and the majority presented an activated CD69^+^ phenotype. In peripheral blood activated, CD69^+^ lymphocytes were absent (not shown).

### Functional characterisation of lymphocytes

The proliferative responses of the cell populations were characterised by time course ^3^H-Thymidine incorporation assays using homogenised denatured autologous tumour cell extracts as antigen sources (Figure 1A and B right panels and Table 2). TILs did not proliferate in any of the investigated patients. By contrast, SN lymphocytes from eight patients classified as Duke's B proliferated in an antigen dependent manner against autologous tumour extract (Figure 1A, right panel and Table 2) with an average peak proliferation of 24567±6473 cpm (average±s.e.m.). Peak proliferation was most frequently observed on day 6. In patient no 5, thymidine incorporation was not performed. SN lymphocytes from five out of six metastatic nodes (patients no 10–12, 14 and 15) responded poorly, with an average proliferation of 2141±1002(average±s.e.m.) (Figure 1B, right panel). From one Duke's C patient (no 13) SN lymphocytes proliferated vigorously when stimulated with autologous tumour extract (30 469 cpm) and also exhibited a high spontaneous proliferation (Table 2). In addition, in two Duke's B patients the reactivity against a known colon cancer antigen, carcinoembryonic antigen (CEA) was tested. However, stimulation of SN lymphocytes with 100 *μ*g ml^−1^ of CEA did not result in proliferation (data not shown). In nonsentinel nodes proliferation upon stimulation with tumour extract was not detected, with the exception of three patients (no 6, 7 and 8) (Table 2). In PBLs the addition of tumour antigen did not result in vigorous proliferation in any case, but responses could be seen in patients no 7, 13 and 14. In the nonsentinel node in patient 11 and in SN lymphocytes from patient 12, high proliferation indexes were obtained (Table 2). They represent single-point measurements, lacking the characteristic dose–response and/or kinetics of antigen-driven proliferation.

**Figure 2:**
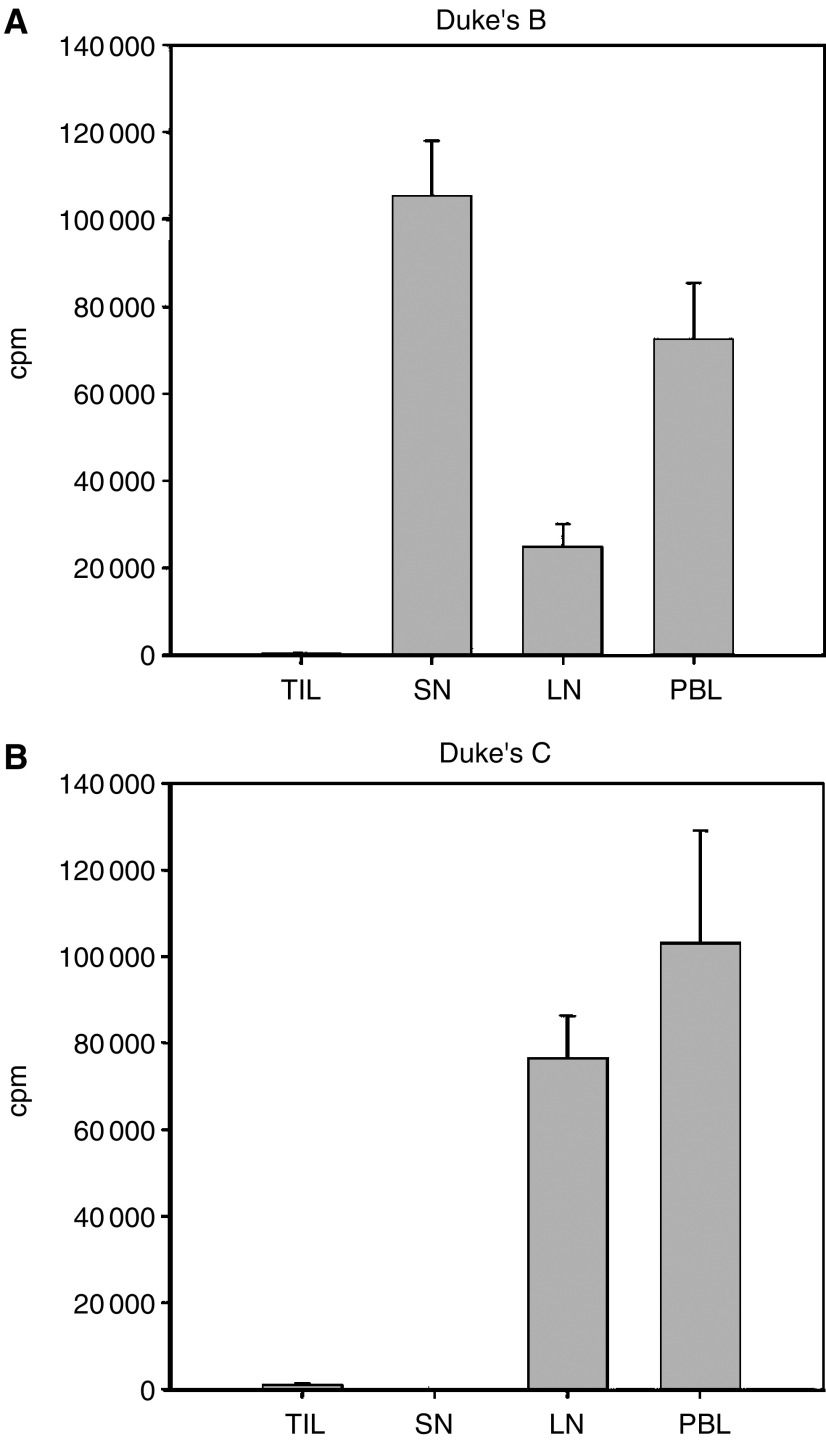
Proliferative responses against Con A. Tumour infiltrating lymphocytes (TIL), cells from sentinel node (SN), from nonsentinel lymph nodes (LN) and peripheral blood leucocytes (PBL), obtained from 15 colon cancer patients were investigated with ^3^H-Thymidine incorporation upon stimulation with 10 *μ*g ml^−1^ of ConA. Data from patient no 2 with a Duke's B tumour (**A**) and patient no 11 with a Duke's C (**B**). Error bars indicate s.e.m.

To further investigate the functional state of the lymphocytes, T cells were stimulated with Con A which by-pass the antigen-specific activation through the T-cell receptor. TILs were unresponsive to Con A stimulation, as were SN lymphocytes from Duke's C patients no 11–12. SN lymphocytes from the remaining patients proliferated vigorously, as did PBLs and nonsentinel node lymphocytes from both C and D patients (Figure 2A and B).

**Figure 3:**
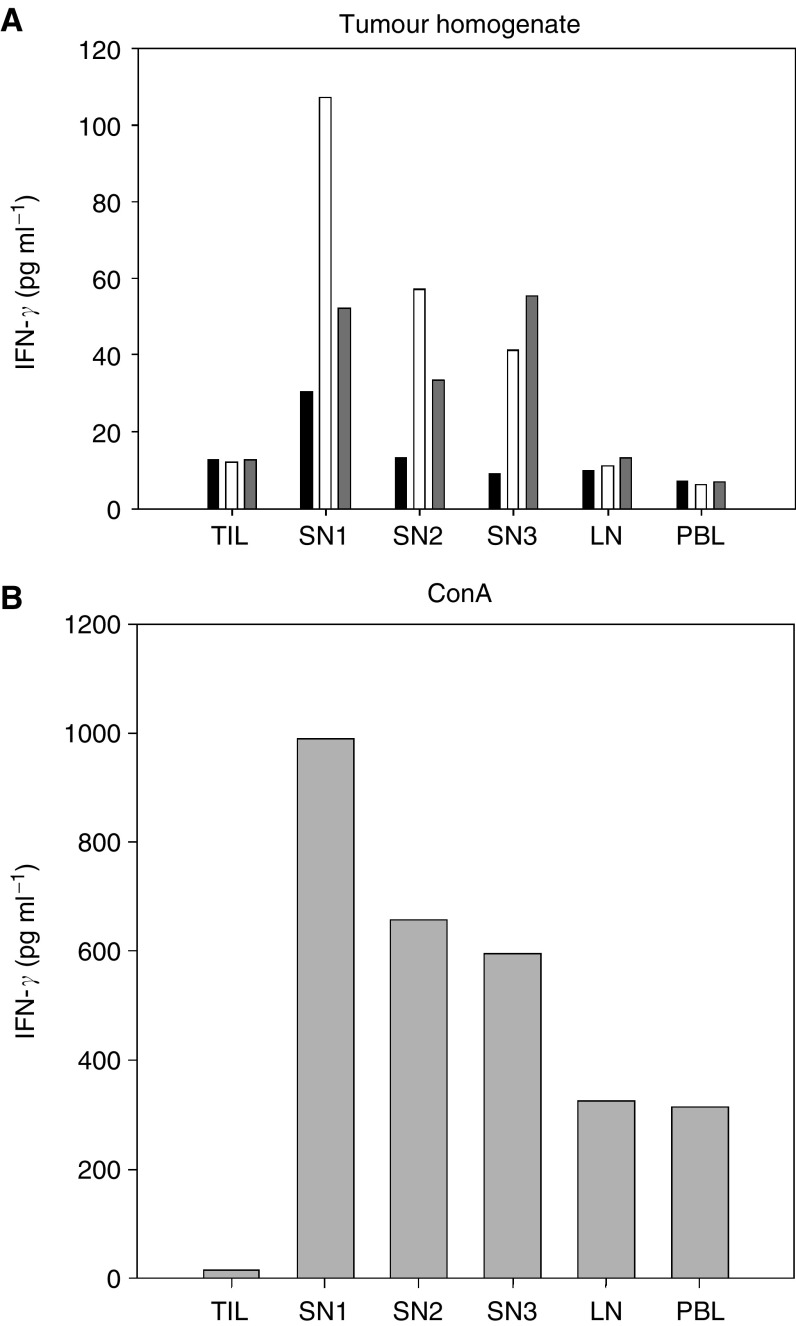
Stimulation with autologous tumour homogenate elicits IFN-*γ* production in lymphocytes from sentinel node. In six of 15 patients, IFN-*γ* secretory responses were investigated with ELISA on culture supernatants. Tumour infiltrating lymphocytes (TIL), lymphocytes from sentinel nodes (SN1-3), from nonsentinel node (LN) and in peripheral blood leucocytes (PBL) from patient no 5, were cultured with medium alone (black bars), autologous tumour homogenate diluted 1/100 (open bars), 1/10 (grey bars) (**A**) or ConA 10 *μ*g ml^−1^ (**B**). The IFN-*γ* production was measured in pooled triplicates of supernatants collected at day 5.

The ability of the lymphocytes to produce IFN-*γ* when stimulated with antigen or with Con A was investigated in six patients, no 4, 5, 8, 13–15 (Table 3). Tumour-infiltrating lymphocytes from Duke's B patient no 4 secreted high amounts of IFN-*γ* when stimulated with Con A (717 pg ml^−1^) and five times the background level upon addition of tumour extract, in spite of their inability to respond in the proliferation assay. Tumour-infiltrating lymphocytes from patient 5 (Duke's B) (Figure 3) and patient 15 (Duke's D) did not produce IFN-*γ* when stimulated with tumour homogenate, nor when they were stimulated with Con A (data not shown). In patients 8, 13 and 14 (Duke's C), the amount of TILs obtained, did not suffice for the IFN-*γ* ELISA.

**Figure 4:**
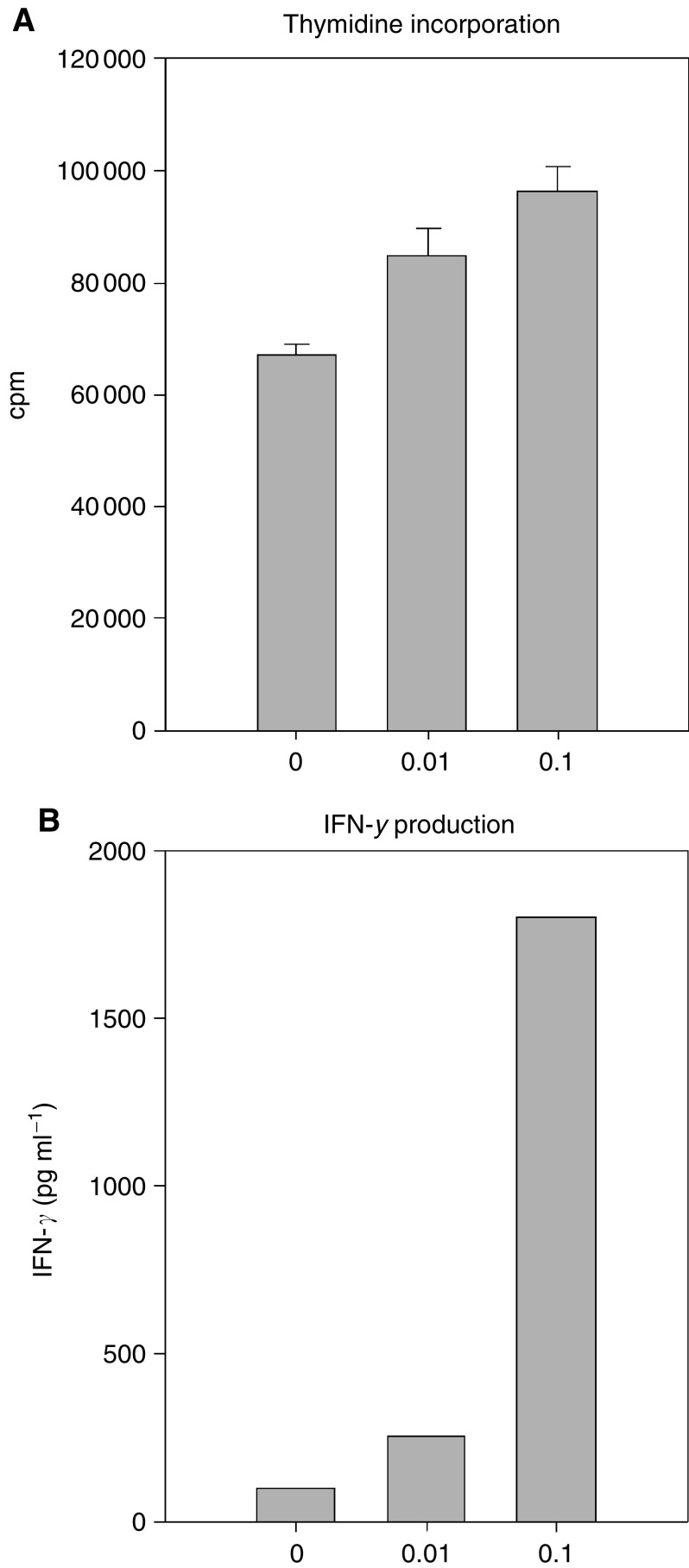
High spontaneous proliferation and specific IFN-*γ* production upon addition of tumour homogenate in cells from a sentinel node in a case of Duke's C. In six of 15 patients, IFN-*γ* secretory responses investigated with ELISAs on culture supernatants. A single cell suspension from the sentinel node from patient no 13 was incubated with 10- and 100-fold dilutions of autologous tumour homogenate in a time course proliferation assay. Cells were pulsed with 1 *μC*_i_^3^H-Thymidine, 18 h before harvesting. Error bars indicate s.e.m. (**A**). The corresponding IFN-*γ* production by the sentinel node acquired lymphocytes was measured in an ELISA on pooled triplicates of supernatant collected day 7 (**B**).

SN lymphocytes from Duke's B patients no 4, 5 (Figure 3A) and 8, and from Duke's C patient no 13 (Figure 4B) secreted IFN-*γ* 4–38 times above background level when stimulated with autologous tumour homogenate. In patients no 14 and 15 SN lymphocytes did not produce IFN-*γ* above background, upon stimulation with tumour homogenate. Lymphocytes from nonsentinel nodes did not respond with any IFN-*γ* secretion above background levels (Figure 3A), with the exception of patient no 8, where the secretion was 30 times the background level. In patients no 4 and 8 secretory responses of four to five times background level were seen in PBLs. SN-, nonsentinel lymphocytes and PBLs displayed a vigorous secretory response when stimulated with Con A (data not shown).

## DISCUSSION

In this study we identify a localised immune response against autologous tumour extract in patients with colon cancer, by using an injection technique allowing for the identification of the tumour draining sentinel node (Figure 1A, right panel, Table 1). SN lymphocytes have been endogenously sensitised towards tumour-derived antigens presented by professional antigen presenting cells and represent a naturally *in vivo* expanded population of cells that can be further multiplied and possibly used for immunotherapy.

Proliferation in sentinel nodes, displaying dose dependence and/or kinetics of antigen dependence, was present in a majority of patients (Figure 1A, right panel, Table 2). Responses were of varying magnitude and tended to be weaker or absent in patients with metastatic sentinel nodes (Table 2 and Figure 1B). We have recently described proliferative responses against the autologous tumour in sentinel nodes draining urinary bladder cancers (Marits *et al*, 2006), further supporting the immunological value of the sentinel node detection method. In the three Duke's B patients no 6–8 dose-dependent proliferation was also detected in nonsentinel lymph nodes. In patients 6 and 8 these nodes were located on the predicted lymphatic drainage pathway from the tumour. In patient 7, the exact anatomical location of the investigated nonsentinel lymph node could not be determined. However, all the 22 investigated lymph nodes in the pathological specimen were enlarged and displayed histological evidence of immune reactivity.

In six of the patients the IFN-*γ* secretory response was investigated (Table 3). The IFN-*γ* response correlated closely with the proliferative responses, with a few exceptions: In patient 4, TILs did produce IFN-*γ*, but they did not proliferate in response to the autologous tumour. The same applied to PBLs from this patient and PBLs from patient 8. This possibly reflects a different state of effector differentiation in these cells than in lymph node acquired cells, exemplified by Duke's C patient no 13. Here, SN lymphocytes displayed vigorous, spontaneous proliferation which could be further augmented by the addition of tumour homogenate, possibly indicating an ongoing expansion of tumour-specific effector cells. By contrast, IFN-*γ* secretion was strongly inducible by antigen stimulation but undetectable in unstimulated cultures (Figure 4). Factors influencing long-term tumour free survival following surgical resection of colon cancers are incompletely understood. Histological parameters indicating a T-cell mediated immune response in mesocolic lymph nodes have been found to correlate with increased survival rates (Patt *et al*, 1975). It is tempting to speculate that the strong immune response in patient no 13 is a predictor of survival. This will be addressed when follow-up data from the patients is available.

TILs displayed an activated CD69^+^ phenotype, similar to SN lymphocytes (Figure 1A and B middle panels), but when tested against autologous tumour antigens (Figure 2A and B right panels) or Con A (Figure 2A) they did not proliferate. In two metastatic sentinel nodes, from patients no 11 and 12, the response against ConA was also blunted, indicating a state of tumour-induced immunosuppression. Some investigators (Hoon *et al*, 1987; O’Sullivan *et al*, 1996) have observed immunosuppression in regional lymph nodes also in the absence of lymph node metastases. This contrasts with our data, where SN lymphocytes in Duke's B patients responded adequately to the mitogen ConA (Figure 2B). This may reflect differences in the biology of the primary tumour, as indicated by the study by O’Sullivan *et al* (1996), who found evidence for immunosuppression in lymph nodes draining squamous cell carcinomas of the oesophagus but not in adenocarcinomas. Secretion of immunosuppressive factors from CRC cell lines has been reported previously (Luo *et al*, 1997). Fresh CRC specimens have been shown to express indoleamine 2,3-dioxygenase (IDO), an enzyme, which catalyses tryptophan degradation. Tryptophan depletion causes proliferative arrest of T cells and hampers local antitumour immunity in mouse models (Uyttenhove *et al*, 2003). In addition, chemokine-mediated recruitment of CD4^+^CD25^+^ T regulatory cells to the tumour bed, has recently been described in ovarial cancer (Curiel *et al*, 2004). The influence of CD4^+^CD25^+^ cells on immune responses against human colon cancers is under investigation in the laboratory.

Naturally induced, tumour-reactive T cells as measured with specific cytokine secretion in response to tumour antigens have been identified in blood in a subset of CRC patients albeit at a low frequency (Nagorsen *et al*, 2000). We detected proliferation in peripheral blood in the three Duke's C patients no 7, 13 and 14 (Table 2), while PBLs from the other 12 patients were unresponsive. This is consistent with that the majority of these cells have not encountered tumour antigens and thus the precursor frequency of T cells in peripheral blood with a specific T-cell receptor recognising tumour antigen(s) is low.

In a murine system, tumour sensitised but not fully functional, lymphocytes were identified in tumour draining lymph nodes. These cells could be converted into effector cells by *in vitro* expansion with anti-CD3mAB and IL-2 and were capable of mediating regression of established metastases following adoptive transfer (Yoshizawa *et al*, 1991). In human CRC; adoptive transfer of *in vitro* expanded lymph node cells, has been attempted in patients with advanced metastatic disease (Kim *et al*, 1999). The lymph nodes were identified by radioimmuno-guided surgery (RIGS) utilising a ^125^I labelled monoclonal antibody against a mucin expressed by carcinomas, consequently dependent on the presence of tumour cells or tumour-derived material in the nodes. In this study, one patient achieved a partial response and 4 patients (4/23) minor responses and an overall increase in survival was observed (Kim *et al*, 1999), indicating a role for immunotherapy in colon cancer. In comparison with the present work, we identify the sentinel node(s), which are the first lymph node(s) to drain the primary tumour, without the use of radioactivity or depending on the presence of specific tumour-derived epitopes in the node(s).

In summary, we demonstrate that freshly isolated lymphocytes from sentinel nodes in patients with established colorectal carcinoma mount proliferative responses against autologous tumour extract. These cells have not yet been exposed to the immunosuppressive milieu of the tumour and constitute an *in vivo* expanded source of tumour reactive lymphocytes. Instead of using TIL or peripheral blood lymphocytes, we propose sentinel node-derived lymphocytes as a source of enriched tumour reactive T cells, suitable for adoptive immunotherapy.

## Figures and Tables

**Table 1:** Patient characteristics, location of tumours, staging and investigated lymph nodes

**Patient No**	**Sex**	**Age (year)**	**Tumour site**	**Duke's classification**	**Metastatic nodes/harvest nodes**	**No of SNs obtained**	**Number of cells in SN (× 106)**	**%CD4/%CD8^a^**
1	M	80	Sigmoid	B	0/18	1	27	44/6
2	M	81	Ascending	B	0/22	1	63	52/7
3	F	58	Ascending	B	0/29	1	MI	41/7
4	F	80	Ascending	B	0/5	2	29	65/4
5	M	82	Ascending	B	0/12	4	14	55/14
6	M	66	Descending	B	0/22	3	66	47/5
7	F	73	Transversum	B	0/23	4	26	58/7
8	M	66	Caecum	B	0/19	2	159	35/6
9	M	71	Descending	B	0/25	3	121	43/10
10	M	70	Sigmoid	C	1/14	2	33	55/8
11	F	78	Ascending	C	2/19	2	11	27/58
12	F	75	Ascending	C	4/23	2	36	37/ND
13	F	63	Sigmoid	C	6/20	3	170	41/7
14	F	57	Sigmoid	C	1/17	2	278	41/14
15	F	61	Sigmoid	D	1/13	3	30	34/6

MI=Missing information.

ND=Not done.

a Average percentage of CD4^+^ and CD8^+^ cells in gated SN lymphocytes measured by flow cytometry. Lymphocytes were defined by gating on forward and side scatter characteristics.

**Table 2:** Proliferative responses in lymphocytes from sentinel node, the tumour, nonsentinel node and peripheral blood upon stimulation with autologous tumour extract

	**SN**				**TIL**				**LN**				**PBL**			
**Pat nr**	**BG^a^**	**Peak^b^**	**Peak-BG**	**SI^c^**	**BG^a^**	**Peak^b^**	**Peak-BG**	**SI^c^**	**BG^a^**	**Peak^b^**	**Peak-BG**	**SI^c^**	**BG^a^**	**Peak^b^**	**Peak-BG**	**SI^c^**
1	9672	26 289	16 617	2,7		ND			4299	7141	2842	1,7	2496	4731	2235	1,9
2	4049	14 559	10 510	3,6	382	1485	1103	3,9	818	509	—	0,6	2836	3301	465	1,2
3	1207	15 125	13 918	12,5	196	1022	826	5,2	2201	3134	933	1,4	553	288	—	0,5
4	1104	43 446	42 342	39,4	186	546	360	2,9		ND			4570	2536	—	0,6
5		ND				ND				ND				ND		
6	9810	55 430	45 620	5,7	1037	2482	1445	2,4	9495	35 032	25 537	3,7	1592	4245	2653	2,7
7	3555	32 141	28 586	9,0		ND			2155	79 629	77 474	37,0	1360	23 257	21 897	17,1
8	1051	6270	5219	6,0		ND			5400	110 655	105 255	20,5	2168	3620	1452	1,7
9	449	3283	2833	7,3		ND				ND			1092	1377	285	1,3
10	488	1218	730	2,5		ND			313	1109	796	3,5	50	52	2	1,0
11	127	238	111	1,9	262	511	249	2,0	410	3878	3468	9,5^d^	645	627	—	1,0
12	341	3848	3507	11,3^d^	423	1237	814	2,9	1009	3223	2214	3,2	180	797	617	4,4
13	66901	97 370	30 469	1,5		ND				ND			27 333	56 664	29331	2,1
14	3328	4506	1178	1,4		ND				ND			796	13 331	12535	16,7
15	398	248	—	0,6	106	131	25	1,2	215	131	—	0,6	120	126	6	1,1

All values are given in counts per minute (cpm) except the Stimulation Index (SI).

SN, sentinel node; TIL, tumour infiltrating lymphocytes; LN, nonsentinel lymph nodes; PBL, peripheral blood leukocytes.

ND=Not determined.

—=Below background value.

a BG=Background, proliferation in complete medium, mean value from triplicate.

b Peak=The highest proliferation upon stimulation with autologous tumour extract, mean value from triplicate.

c SI=stimulation index=peak/BG.

d Single high measurements without dose–response or antigen dependent kinetics.

**Table 3:** Production of IFN-*γ* upon stimulation with autologous tumour extract.

	**SN**			**TIL**			**LN**			**PBL**		
**Pat nr**	**BG^a^ (pg ml^−1^)**	**Peak^b^ (pg ml^−1^)**	**SI^c^**	**BG^a^ (pg ml^−1^)**	**Peak^b^ (pg ml^−1^)**	**SI^c^**	**BG^a^ (pg ml^−1^)**	**Peak^b^ (pg ml^−1^)**	**SI^c^**	**BG^a^ (pg ml^−1^)**	**Peak^b^ (pg ml^−1^)**	**SI^c^**
4	12	463	38,6	26	120	4,6	8	12	1,5	11	12	1,1
5	30	107	3,7	13	13	1	10	13	1,3	7	7	1,0
8	14	82	5,9	ND	ND	ND	62	688	11,0	16	93	5,8
13	97	1806	18,6	ND	ND	ND	ND	ND	ND	ND	ND	ND
14	6	14	2,3	ND	ND	ND	ND	ND	ND	ND	ND	ND
15	175	94	0,5	24	29	1,2	16	23	1,4	20	17	0,9

SN, sentinel node; TIL, tumour infiltrating lymphocytes; LN, nonsentinel lymph nodes; PBL, peripheral blood leukocytes.

ND=Not determined.

a BG=Background, secretion of IFN-γ when cultured in complete medium alone, value from pooled triplicates.

b The highest amount of IFN-*γ* secreted upon stimulation with autologous tumour extract, value from pooled triplicates.

c SI=stimulation index=peak/BG.
